# Construct validity, reliability and measurement invariance of the intervention usability scale - insights from two psychological interventions in primary health care

**DOI:** 10.1186/s43058-026-00951-w

**Published:** 2026-05-22

**Authors:** Kasperi Mikkonen, Markus S. Hannukkala, Jari Lahti, Samuli I. Saarni, Suoma E. Saarni

**Affiliations:** 1 Department of Psychiatry, Helsinki University Hospital, Helsinki, Finland; 2 Department of Psychology, Faculty of Medicine, University of Helsinki, Helsinki, Finland; 3 Pediatric Neurology, Helsinki University Central Hospital and University of Helsinki, Helsinki, Finland; 4 Folkhälsan Research Centre, Helsinki, Finland; 5 Faculty of Medicine and Health Technology, Tampere University, Tampere, Finland; 6 Department of Psychiatry, The Wellbeing Services County of Pirkanmaa, Tampere, Finland; 7 Family and Social Services, Psychiatry, Wellbeing Services County of Päijät-Häme, Lahti, Finland

**Keywords:** Usability assessment, Implementation measures, Psychometric evaluation, Psychological interventions

## Abstract

**Background:**

Usability has been proposed as a critical factor influencing the implementation of psychological interventions in routine practice. The Intervention Usability Scale (IUS) has previously been adapted from the System Usability Scale to assess the perceived usability of psychological interventions. However, the construct validity, reliability and theoretical consistency of the IUS remain unclear. This study evaluates the psychometric properties of the Finnish version of the IUS in two cognitive–behavioral therapy-based interventions delivered in primary healthcare.

**Methods:**

A translated Finnish version of the IUS was administered to healthcare professionals trained in either low-intensity guided self-help (*n* = 921) or high-intensity face-to-face cognitive–behavioral therapy programs (*n* = 455). Exploratory and confirmatory factor analyses were conducted to examine the underlying structure of the IUS. Configural, metric and scalar invariance was assessed across multiple demographic subgroups. Reliability was assessed using McDonald’s Omega.

**Results:**

Exploratory analyses suggested a three-factor structure, contradicting previous findings supporting a unidimensional or two-factor models. Confirmatory factor analysis in the guided self-help dataset showed moderate model fit after treating identified issues with correlated residuals and item cross-loadings. Measurement invariance was established across all assessed demographic characteristics. However, the identified factor structure did not replicate in the face-to-face cognitive–behavioral therapy dataset, raising concerns about the generalizability of the measure across different interventions. Reliability estimates were acceptable in the guided self-help dataset but weaker in the face-to-face cognitive–behavioral therapy dataset.

**Conclusions:**

The findings suggest that the IUS does not robustly measure usability as previously proposed. The measure’s factor structure varies across different interventions, and its alignment with theoretical models of usability is unclear. These results question the applicability of the IUS for assessing usability in psychological interventions. If usability is to be measured in this context, a domain-specific approach may be necessary. A roadmap for further measure development is outlined. Until further validation is conducted, the use of the IUS for assessing psychological interventions to guide intervention implementation is not recommended.

**Supplementary information:**

The online version contains supplementary material available at 10.1186/s43058-026-00951-w.

**Table:** 

Contributions to the literature	
• The article expands understanding of the conceptualization and measurement of usability - a proposed critical factor in the implementation of psychological interventions - from both theoretical and empirical viewpoints.	
• The article refines recommendation for measuring usability, emphasizing that further research and development is needed before the measure can be confidently applied.	
• The article demonstrates how the construct validity, reliability, and measurement invariance of implementation measures can be robustly examined, providing a methodological framework for future research.	

## Background

Mental disorders pose a global public health problem [1]. Despite extensive research on effective treatments for mental disorders, access to care remains inadequate worldwide [2, 3]. The discourse on the implementation of evidence-based interventions has gained significant attention recently, as the degree to which these interventions are implemented plays a key role in realizing their potential health and well-being benefits in routine practice [4]. A myriad of potential determinants of implementation success have been proposed, including organizational features and individual-level factors. The perceived usability of an intervention has been theorized as an implementation mechanism relevant to both perceptual (e.g., feasibility, acceptability, appropriateness) and behavioral (e.g., adoption and sustainment) implementation outcomes [5].

The conceptualization of usability often emerges from the interplay between three contributors: the user, the surrounding environment, and the system design [6]. Usability in its modern usage has been used since as early as the 1980s, especially in the context of software engineering and human‒computer interaction (HCI). The birth of the modern usability profession can be traced back to the work by Whiteside, Bennett and Holtzblatt at IBM (1988) on the topic of usability engineering and the book ‘The Psychology of Everyday Things’ (later ‘The Design of Everyday Things’) by Donald Norman in the same year [7, 8]. In the latter, the concept of usability is presented as a means to reduce the ‘gulf of execution’, which refers to the gap between a user’s goal for action and the means to reach that goal. Usability can be seen as an attempt to bridge this gap by removing roadblocks and extra steps that hinder the execution of a specific task to reach certain goals [8, 9]. In subsequent work, the theoretical conceptualization of usability often highlights the multifaceted structure of the concept. A foundational theoretical model of usability recognizes the following facets of usability: learnability (how easy it is to accomplish tasks first when encountering the system), efficiency (how quickly tasks can be performed), memorability (how to re-establish proficiency after a period of not using), errors (how often errors occur, how severe they are and how to recover from errors), and satisfaction (how pleasant it is to use the system) [10]. The International Organization of Standardization (ISO) currently defines usability generically as the ‘extent to which a system, product or service can be used by specified users to achieve specified goals with effectiveness, efficiency and satisfaction in a specified context of use’ [11].

The generic approach to conceptualizing usability has also been called into question. The contemporary literature on usability often emphasizes the domain-specific nature of the concept, which influences the facets of usability that are prioritized, how they are measured, and which kinds of usability benchmarks are considered relevant. For example, domains such as in-vehicle information systems, aging populations, autistic populations, electronic health record (EHR) systems and cybersecurity all emphasize different aspects of usability [12]. Usability as a construct has also been viewed as an umbrella construct with multidimensional history- and context-dependent properties that do not point toward unidimensional and clear definitions and that the concept itself warrants actions in terms of either restructuring or abandonment [13].

There are numerous ways to evaluate usability, including inspection methods (e.g., cognitive walkthroughs), testing methods (e.g., performance measurement) and inquiry methods (e.g., questionnaires) [14]. Researchers have addressed the importance of standardized usability questionnaires for practitioners employing traditional methods of standardized questionnaires on the basis of their psychometric properties [15, 16]. One of the most commonly used questionnaires for assessing perceived usability is the system usability scale (SUS). Originally, the SUS was developed as a unidimensional tool to provide a single, comprehensive measure of perceived usability. However, evaluations of the construct validity of the SUS have yielded mixed results. These range from support for the originally proposed unifactorial structure to two alternative two-factor models: one distinguishing between positive- and negative-toned items, and the other identifying factors labeled as ‘usable’ and learnable’ [17]. In previous research, the SUS was found to correspond closely to other measures of perceived usability, including ‘the Usability Metric for User Experience’ (UMUX), UMUX-LITE (a shorter version of UMUX) and ‘the Computer System Usability Questionnaire’ (CSUQ) [18, 19].

In the context of the implementation of psychological interventions, numerous usability issues have been recognized, including but not limited to complexity, limited available time, incompatibility with provider preferences or practices, incompatibility with existing workflows, insufficient customization to clients and provider buy-in (trust) [20]. To measure usability in the context of psychological interventions, the original SUS has been modified to ‘the Intervention Usability Scale’ by changing the word ‘system’ to ‘implementation’ [21]. Lyon et al. assessed the psychometric properties of the IUS in the context of motivational interviewing and reported that a two-factor structure best fit the data, concluding that the IUS is a suitable instrument for assessing the usability of complex health interventions. A complex intervention was described as an intervention with several interacting components. Following a previously discussed structure related to the SUS measure, the authors labeled the factors as ‘usable’ and ‘learnable’. However, the authors did not provide a theoretical explanation for why the psychometric properties of the IUS align with those of the SUS. Furthermore, they did not extensively report fit indices related to their model but concluded that the IUS demonstrated good psychometric quality and a structure consistent with prior research. These findings, along with the theoretical ambiguity of the usability concept and its applicability in this context, highlight the importance of replication and further investigation.

In this paper, we describe the translation process of a Finnish version of the IUS and examine its construct validity, reliability, and measurement invariance. The IUS is examined in the context of two interventions based on cognitive‒behavioral therapy (CBT), which are used by primary healthcare professionals working with mental health problems. The aim is to examine the psychometric properties of the IUS and align it with existing theoretical frameworks of usability. Furthermore, we aim to enhance the understanding of the IUS and evaluate the applicability of a generic measure of usability in the context of psychological interventions. The study answers the following research questions:

1. Does the Finnish version of the IUS replicate the previous findings of a unifactorial or two-factor structure in two datasets concerning the usability of two separate cognitive‒behavioral interventions? How well do the previously recognized models fit the data and are the findings congruent between datasets?
2. What factor structure provides the best fit to the data, and how does it align with the theoretical basis of usability?
3. What is the level of measurement invariance of the Finnish version of the IUS regarding the demographic variables gathered in the datasets?

## Methods

### Measures

The IUS is an adapted version of the 10-item SUS where the term ‘system’ is replaced with ‘intervention’ [21]. In this study, a translated Finnish version of the IUS was evaluated. The IUS consists of 10 items, of which five are positively toned and five are negatively toned. The answers are given on a five-point Likert scale (0 = Strongly disagree, 4 = Strongly agree). For negatively worded items, scores are reversed-coded prior to summation. No respondents were excluded based on within-scale response inconsistency, as the IUS does not include established inconsistency indices or paired items that would allow reliable identification of contradictory responding at the individual level. All analyses were therefore conducted on the full dataset following standard scoring procedures of the SUS, and by extension the IUS. The total score is calculated by multiplying the summed item scores by 2.5, yielding a total score ranging from 0 to 100 and. This transformation rescales the original 40-point raw score (10 items × maximum contribution of 4 points) to a standardized 0–100 metric, facilitating interpretation and comparability across studies and contexts.

The translation process of the IUS was as follows: three separate versions from English to Finnish were used (one translated by a subject matter expert (KM), one by a professional translator, and one using a generative AI agent). A working group (including KM and SES from the authors) cross-evaluated the different versions to formulate a version with the best balance between understandable language and equivalence with the original. Furthermore, as no official Finnish translations of the SUS were available, a small number of unofficial Finnish translations available online were consulted to inform the translation process (e.g., to provide alternative wording and sentence structure). These unofficial translations were used solely as a complementary reference when finalizing the wording of the translated items. An independent back translation was ordered and used to assess the congruence between the translated version and the original version which was assessed by KM and SES. Furthermore, the translated version of the IUS was piloted within the training programs conducted at Helsinki University Hospital which enabled the identification of potential practical or comprehension-related issues when using the instrument in its intended context.

### Participants and procedures

The study participants were healthcare professionals from various professional backgrounds who had completed or were currently participating in two different training programs focused on psychological interventions for common mental health problems and disorders. All participants work or have worked in the time of training in a publicly funded mental health context in Finland. The participants were invited to a survey in which they evaluated the usability of the intervention they were trained in. The survey was sent to trainees taking part in a low-intensity guided self-help training program and in a longer training program in a higher-intensity face-to-face therapy modality. It is important to note that the interventions included in this study differ in intensity and structure; however, both comprise multiple interacting components characteristic of complex interventions and are implemented within the same service context (primary care). Moreover, given that motivational interviewing – the reference point for intervention complexity in the original IUS publication, consisting of multiple interacting techniques and applicable across a range of clinical targets – was used to justify the scale’s applicability, it was judged reasonable to assess the usability of both interventions despite these differences. These form the two datasets used in the study. Each participant provided informed consent at the beginning of the survey. The study was approved by the ethical committee of Helsinki University Hospital (HUS322/2024) and was performed in accordance with the Declaration of Helsinki.

#### Dataset 1: Usability of guided self-help

Guided self-help (GSH) is a 1–5 session intervention based on cognitive‒behavioral therapy aimed at treating symptoms of common mental health disorders [22]. The training program is 5–10 hours long and tailored on the basis of the needs of the target population (e.g., parents, adolescents). The training incorporates an e-learning course as well as skill workshops. A total of 921 (15%) respondents out of the 6 335 invited participants were enrolled in the survey. Of these, 72% (663/921) had completed the training, 28% (256/921) were currently in training, and 59% (543/921) had previous training in psychological interventions. The demographic information of this dataset can be viewed in Table 1. The GSH data were split into exploratory and confirmatory datasets via stratified sampling to carry out the necessary analyses regarding the construct validity of the measure. The following variables were used to guide the stratified sampling process to ensure representativeness and balance across the subsets created: gender, age, profession and education level. To confirm that the background variables were balanced across the exploratory and confirmatory datasets, proportional distributions of gender, age, profession, and education were calculated for each subset. These proportions were compared to verify representativeness and ensure the integrity of the stratification process. The random seed was set to ‘123’ to allow for exact replication of the split.

**Table 1:** Demographic characteristics of the study samples

	Dataset 1 (GSH) (*N* = 921)	Dataset 2 (fCBT) (*N* = 455)
**Gender**		
Female (%) Male (%) Other (%) Prefer not to say (%)	92.0 6.4 0 1.6	86.9 11.3 0.7 1.1
**Age**		
Mean SD 18–25(%) 26–35(%) 36–45(%) 46–55(%) 56–65(%) 66+ (%)	45.8 10.3 1.1 17.7 27.9 33.6 19.3 0.3	44.8 9.7 1.1 18.8 30.8 32.6 16.7 0.0
**Profession**		
Nurse (%) Social worker (%) Psychologist (%) Doctor (%) Other (%)	56.7 22.1 8.1 0.2 12.1	84.1 4.0 6.6 1.6 3.8
**Education-level**		
Basic education (%) Upper secondary education (%) Lower university (%) Higher university level (%) Doctoral (%)	0.7 9.7 61.2 28.2 0.2	0.4 10.0 69.5 20.1 0.0
**Working experience (years)**		
Mean SD 0-1 (%) 2-5 (%) 6-10 (%) 11-15 (%) 16-20 (%) 21+ (%)	17.7 10.1 0.9 13.2 15.3 17.5 17.0 36.2	17.0 9.6 1.0 12.0 19.2 16.6 17.3 33.9

#### Dataset 2: Usability of face-to-face cognitive‒behavioral therapy

Face-to-face cognitive‒behavioral therapy (fCBT) is a 5–10 session intervention aimed at treating symptoms of common mental health disorders. The training program is 1-year-long (15 ECTs), includes treatment modalities for multiple mental disorders and incorporates an e-learning course, skills training in supervision and clinical practice. A total of 455 respondents (35%) out of 1 308 invited participants were enrolled in the survey. Of these, 45% (205/455) had completed the training, 55% (250/455) were currently in training, and 59% (268/455) had previous training psychological interventions (Table 1).

### Statistical analyses

For all analyses, we used listwise deletion, meaning that any case (row) with missing data for the variable needed for a given analysis was removed.

All analyses were conducted in R 4.4.2. Data manipulation and preparation were performed using the tidyverse [23], including dplyr [24]. Exploratory factor analysis, parallel analysis, and descriptive statistics were conducted using the psych package [25]. The optimal number of factors was further evaluated using scree plot and parallel analysis procedures implemented in the nfactors package [26]. Oblimin rotation in exploratory factor analysis was performed using GPArotation [27]. Confirmatory factor analysis and measurement invariance testing were conducted using lavaan [28], with semTools [29] used to compute reliability estimates (McDonald’s omega) and to compare measurement invariance models. Factor structures were visualized using semPlot [30].

The statistical procedure was performed in four steps:

#### Step 1: Exploratory factor analysis (EFA) with split dataset 1

As a first step, we calculated a scree plot. A scree plot is a graphical representation of the eigenvalues associated with the factors or components of a dataset, plotted in descending order. Eigenvalues reflect the amount of variance explained by each factor. To construct a scree plot, we extracted the eigenvalues from a polychoric correlation matrix. We interpreted the scree plot qualitatively by identifying the ‘elbow’ or the point where the slope of the plot levels off. This or these inflection points suggest the optimal number of factors to retain, as the factors beyond the elbow contribute minimal variance and are more likely due to random error or noise.

To complement the scree plot, we conducted a parallel analysis. Parallel analysis compares the observed eigenvalues from the dataset with those generated from randomly simulated data with the same dimensions [31]. Factors are retained only if their observed eigenvalues exceed the corresponding eigenvalues from the random data. Together, the scree plot and parallel analysis provide evidence for determining the dimensionality of the IUS, ensuring that a meaningful quantity of factors is identified. As Li and Jahng conclude from their simulations, the number of factors suggested by parallel analysis should not be considered a fixed estimate but rather a starting point around which a suitable number of factors can be determined [31].

We assessed the dimensionality of the IUS via exploratory factor analysis (EFA) [32] with a weighted least squares estimator with Oblimin rotation to permit factor intercorrelation. We selected the best model by considering fit indices, such as the root mean square of the residuals (RMSR), the root mean square error of approximation (RMSEA), and the Tucker‒Lewis index (TLI), alongside the inspection of factor loadings and their theoretical relevance.

#### Step 2: Confirmatory factor analysis (CFA) with the remaining half of dataset 1

Following the exploratory analyses, we evaluated the structural validity of the IUS via confirmatory factor analysis (CFA) under the framework of the weighted least squares mean and variance adjusted (WLSMV) estimation, with the remaining half of the split dataset. We chose the WLSMV estimator because of its suitability for ordinal data and its ability to provide robust standard errors and model fit indices with non-normal data [33]. In this study, model-data fit was assessed with a chi-square test (χ^2^), which tests the null hypothesis that the covariance matrix of the model implied does not significantly differ from the matrix of observed covariances. This study utilized approximate fit indices, including the root mean square error of approximation (RMSEA) [34], standardized root means square residual (SRMR), comparative fit index (CFI) [35] and Tucker‒Lewis index [36]. Fit was considered satisfactory when the CFI and TLI were close to or greater than 0.95, the RMSEA was lower than or equal to 0.06, and the SRMR was lower than or equal to 0.08 [37]. We evaluated approximate fit indices via standardized and scaled values and did not use them as strict cutoff values but rather as indicators of misfit. When the $\chi$2 value was significant and/or the approximate fit indices were unsatisfactory, we evaluated the expected parameter change statistics [38] and correlation residuals. We considered normalized correlation residuals >|0.1| as troublesome for model fit [39]. We thoughtfully inspected computed factor models for possible indications of misfit, including convergence problems, improbable values of estimated parameters or standardized values, collinearity, inflated standard errors, and negative variances [40].

In this study, the reliability of the IUS was examined in tandem with CFA via McDonald’s omega values. McDonald’s omega is more general than Cronbach’s alpha, requires fewer restrictive assumptions, and can be interpreted in ways similar to those of alpha [41]. Omega values aim to express the proportion of factor variance in the latent structures of the survey [42]. In this study, omega values greater than 0.7 were considered acceptable, and values higher than 0.8 were considered good.

#### Step 3: Assessing the measurement invariance with dataset 1

MI evaluates whether the IUS factor structure functions equivalently across different respondent groups. We tested for three levels of invariance: configural invariance to determine if the factor structure is consistent across groups, metric invariance to assess the comparability of item loadings across groups, and scalar invariance to examine whether group differences in latent factor means can be meaningfully interpreted [43].

We assessed model fit during the various phases of MI evaluation via several indices, as suggested by Chen [44]. In addition to the χ^2^ statistic, we defined significant changes in fit indices as follows: a decrease of −0.01 in CFI (ΔCFI), an increase of 0.015 in RMSEA (ΔRMSEA), and an increase of 0.030 (metric invariance) or 0.015 (scalar invariance) in SRMR (ΔSRMR). We noted the χ^2^ statistic but did not heavily weight it, as its *p* value is sensitive to large sample sizes.

We examined MI via the CFA model identified in the earlier step and applied it to the dataset across several grouping criteria:

1. Well-being service county: Respondents were randomized into two groups on the basis of the wellbeing service county in which they work.
2. Age: Those below and those above (or equal to) the median age
3. Work experience: Respondents were split into two groups on the basis of the median years of work experience.
4. Previous training in psychological interventions: Respondents were divided into two groups: those who had previous training in psychological interventions and those who had not.

#### Step 4: CFA on dataset 2

We tested the CFA found with dataset 1 using dataset 2 to assess the validity of the model in another intervention. We used the same criteria to assess the model fit.

## Results

Comparisons of the back-translated version with the original English items did not reveal substantive differences in item tone and wording, suggesting that the instructions, response scales, and item content of Finnish version were likely to be interpreted in a manner comparable to the original instrument. Furthermore, no substantial difficulties in the practical use of the IUS were observed during the piloting conducted prior to the study, and no modifications to the translated version were required.

### Step 1: EFA with split dataset 1

**Fig. 1:**
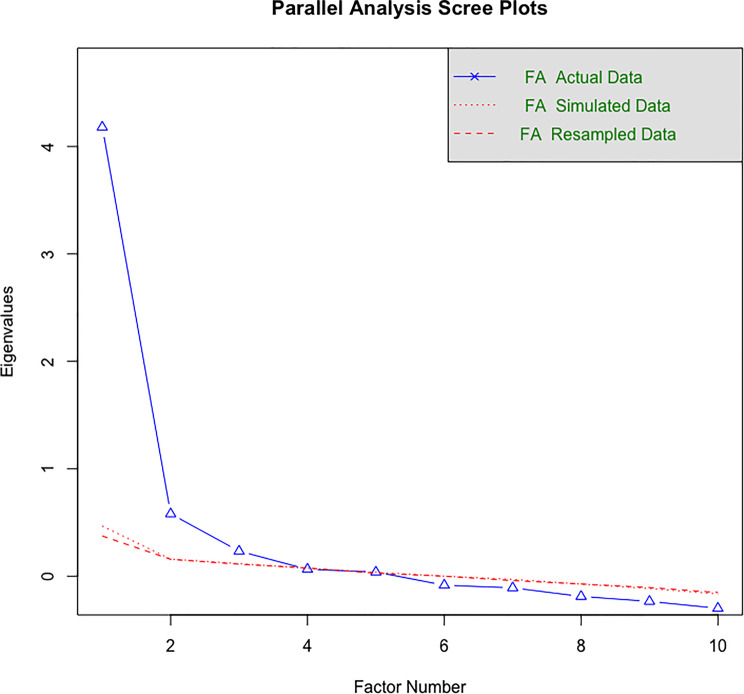
Scree plot displaying the eigenvalues associated with the factors extracted from the dataset

The scree plot revealed two and four factors (Fig. 1), and parallel analysis suggested that three factors should be extracted. Despite the theoretical background of IUS looking after a unifactorial model, this analysis seems to rule out that possibility at this point of our analysis strategy. Considering the previous empirical findings of a two-factor structure and the current results suggesting three or even four plausible factors, solutions with two to four factors were deemed important for further evaluation. Detailed results of the EFA are displayed in Additional File 1.

Solutions with two, three, and four factors were examined and compared on the basis of their statistical fit, variance explained, and theoretical interpretability. The two-factor solution explained 50% of the variance (28 and 22% for Factors 1 and 2, respectively), but cross-loadings were observed for several items, and the RMSR and TLI indicated a less-than-optimal model.

The three-factor solution explained 54% of the variance (23, 13, and 18% across Factors 1, 2, and 3, respectively). Compared with the two-factor solution, the three-factor solution exhibited better overall fit, with an RMSR of 0.02, RMSEA of 0.072, and TLI of 0.941. Items 3, 4 and 9 displayed some cross-loading concerns, and item 7 did not load significantly into any factor. The four-factor solution, while explaining 59% of the variance, yielded diminishing returns in terms of fit, with an RMSEA of 0.08 and a TLI of 0.928. Item 7 was found to be the only item loading to the factor raising concerns about the interpretability of the fourth factor.

On the basis of these findings, no unifactorial solutions could be derived from the dataset. The three-factor solution provided the best balance between statistical fit, parsimony, and theoretical coherence, warranting further examination in confirmatory analyses.

### Step 2: CFA with the remaining data from dataset 1

A three-factor CFA converged with a statistically significant $\chi$^2^ value ($\chi$^2^ = 198.052, df = 32, *p* < 0.001). CFI (=0.948), TLI (=0.927) and SRMR (=0.077) were found close to satisfactory levels, whereas RMSEA (=0.143, 90% CI 0.124–0.163) was found to be inflated. The standardized factor loadings varied from 0.654 to 0.926 (Table 2). All the estimated variances of the items and factors were positive and ranged from 0.143–0.706. Factor correlations were 0.616 between factor 1 and factor 2, 0.880 between factor 1 and factor 3, and 0.510 between factor 2 and factor 3, respectively. Omega values were found to be acceptable: factor 1 was 0.861, factor 2 was 0.737, and factor 3 was 0.799.

**Table 2:** Standardized factor loadings, standard errors, and omega values of the first confirmatory factor analysis

		Standardized factor loading	Standard error
Factor 1 (*ω*=.861)			
	Q6	0.840	
	Q8	0.926	0.038
	Q2	0.836	0.038
Factor 2 (*ω*=.737)			
	Q10	0.701	
	Q4	0.912	0.184
Factor 3 (*ω*=.799)			
	Q5	0.758	
	Q1	0.606	0.059
	Q3	0.890	0.057
	Q7	0.661	0.062
	Q9	0.654	0.061

*ω* = McDonald’s omega

To assess the significance of the test statistic and suboptimal RMSEA, correlation residuals between items were computed. The item pairs [4,1], [4,3], [5,1], [5,4], [8,7], [9,4], [10,5] and [10,9] were found to have excess correlation residuals (Additional File 2). High expected parameter change (EPC) characteristics were found between [Factor 1, 1], [Factor 2, 1], [Factor 2, 9], [10,9] and [5,1].

We attempted the same CFA with several allowed covariances. We allowed the item pairs [4,1], [4,3], [5,1], [5,4], [8,7], [9,4], [10,5] and [10,9] to correlate. This post hoc approach entails a risk of capitalizing on sample-specific characteristics. However, several of the correlated residuals involved item pairs sharing salient wording features – most notably negative item polarity – suggesting that the excess covariance may reflect systematic method variance (e.g. response patterns associated with reverse-worded items), rather than usability-construct-relevant shared variance or purely idiosyncratic error. This model also converged, albeit with a statistically significant χ^2^ value (χ^2^ = 67.050, df = 24, *p* < 0.001). All of the modification indices were found to be closer to the acceptable range (CFI = 0.986, TLI = 0.975, RMSEA = 0.084, 90% CI [0.061, 0.108], SRMR = 0.041). Standardized factor loadings and standard errors were found to be in a similar range as those in the first CFA (Table 3; Additional file 3). Omega values were found to be acceptable: factor 1 was 0.860, factor 2 was 0.736, and factor 3 was 0.764 (Table 3).

**Table 3:** Standardized factor loadings, standard errors, and omega values of the first confirmatory factor analysis

		Standardized factor loading	Standard error
Factor 1 (ω=.860)			
	Q6	0.847	
	Q8	0.910	0.038
	Q2	0.845	0.038
Factor 2 (ω=.736)			
	Q10	0.703	
	Q4	0.910	0.178
Factor 3 (ω=.764)			
	Q5	0.745	
	Q1	0.573	0.059
	Q3	0.899	0.065
	Q7	0.633	0.065
	Q9	0.631	0.064

ω = McDonald’s omega

### Step 3: Measurement invariance

We divided respondents into two groups based on each demographic variable of interest as follows: randomized by wellbeing service county (group 1: *n* = 470; group: 2, *n* = 446), based on age with a median age of 47 years (group 1: *n* = 454; group 2: *n* = 422), based on work experience with a median work experience of 16 years (group 1: *n* = 431; group 2: *n* = 418), based on previous training in psychological interventions (group 1: *n* = 477; group 2: *n* = 431), and based on their training completion status (group 1: *n* = 677; group 2: *n* = 270). Across all analyses, $\chi$^2^ -coefficients were significant, but Δ CFI, Δ RMSEA and Δ SRMR were within acceptable ranges, indicating scalar invariance. Detailed results of measurement invariance analyses can be viewed from Additional File 4.

### Step 4: CFA on dataset 2

A CFA model converged with a significant χ^2^ value (χ^2^ = 116.974, df = 24, *p* < 0.001). The RMSEA was inflated (=0.109, 90% CI [0.088, 0.131]), and the TLI was below adequate (=0.917). CFI was 0.956, and SRMR was 0.055. Standardized factor loadings were lower than those in dataset 1, ranging between 0.501 and 0.872. An inflated standard error was found for factor 2 (item 4 = 0.218). The correlations between factors ranged between 0.441 and 0.882. Factors 1 and 3 had acceptable Omega values (0.744 and 0.714, respectively), but the Omega value for factor 2 was unsatisfactory (0.517). The results of the CFA on Dataset 2 can be viewed from Table 4.

**Table 4:** Standardized factor loadings, standard errors, and omega values of the CFA on dataset 2 (fCBT)

		Standardized factor loading	Standard error
Factor 1 (ω=.744)			
	Q6	0.796	
	Q8	0.818	0.046
	Q2	0.770	0.039
Factor 2 (ω=.517)			
	Q10	0.577	
	Q4	0.747	0.218
Factor 3 (ω=.714)			
	Q5	0.666	
	Q1	0.501	0.057
	Q3	0.872	0.071
	Q7	0.520	0.068
	Q9	0.628	0.063

ω = McDonald’s omega

## Discussion

In this study, we assessed the construct validity and measurement invariance of the Finnish version of the IUS. The IUS is designed to assess how healthcare professionals perceive the usability of a given psychological intervention, providing insight into its practical applicability. We used data from two distinct cognitive‒behavioral interventions trained nationally in Finland in the context of common mental health disorders: low-intensity GSH (1–5 sessions) and more high-intensity fCBT (5–10 sessions). Furthermore, we aim to align the identified factor structure with the theoretical foundations of usability.

The two-factor structure identified in the prior study of the IUS [21] was not replicated in this study. Additionally, our analysis does not support either the unifactorial or two-factor structures previously recognized in studies regarding the SUS, from which the IUS was adapted [17]. Conversely, a three-factor structure was the best fit for exploratory and confirmatory factor analyses in the GSH dataset, but it still showed some issues with global fit indices. The three-factor structure exhibited strong factor loadings and a reasonable CFI/TLI, providing partial support for the model. However, the model converged high correlations between factors, and significant correlation residuals were found between various item pairs. Allowing several item pairs to correlate resulted in a model with a better fit. In particular, items 3 (“I thought the intervention was easy to use”), 4 (“I think that I would need the support of an expert consultant to be able to use this intervention”), and 9 (“I felt very confident using this intervention”) presented communality issues, suggesting tendencies to load onto multiple factors when given the opportunity. Among these items, items 4 and 9 were also found to have high communality in the exploratory factor analysis. Scalar measurement invariance was achieved in this dataset for wellbeing service county, age, work experience, prior training in psychological interventions, and course completion status (completed vs. in training). This suggests that respondents answered the IUS consistently across these demographic variables when evaluating GSH. However, the factor structure that best fit the GSH dataset did not replicate the second fCBT dataset, suggesting that the construct validity of the IUS may differ depending on the intervention being assessed. Reliability analyses further support his concern, as Omega values for the three-factor solution were acceptable in the GSH dataset. However, in the fCBT dataset, omega coefficients were found unsatisfactory. In summary, the construct validity analyses in this study suggest that the IUS may measure multiple constructs, making it unclear what the proposed sum variable, derived from all ten items, represents. Furthermore, the interpretation of this sum variable may differ when different interventions are evaluated.

It is important to distinguish between two types of concerns raised by our findings: statistical misfit and theoretical inadequacy. Statistical misfit refers to technical problems with model specification—cross-loadings, correlated residuals, and suboptimal fit indices—which could potentially be addressed through item revision, model respecification, or refined measurement approaches. Theoretical inadequacy, by contrast, questions whether the construct of usability, as conceptualized in human-computer interaction, meaningfully applies to psychological interventions at all. Our results suggest that both statistical and theoretical concerns are present. At the statistical level, the three-factor model required post hoc modifications to achieve acceptable fit, and several items showed problematic cross-loadings or communality issues. These findings indicate that the IUS items do not cleanly measure distinct constructs as specified. At the theoretical level, the failure of the factor structure to replicate across interventions—and its alignment with item wording rather than usability dimensions—raises deeper questions about whether “usability” is a coherent construct when applied to complex psychological interventions.

The IUS appears to be affected by reverse coding, as negatively and positively worded items do not load on the same factors within the factor structure. Regarding the original SUS, Lewis and Sauro argue that distinctions based on item tone (positive or negative) have little practical or theoretical significance and advocate the use of a single total sum score [45]. Additionally, Lewis [17] reported that a version of the SUS containing only positive items produced mean scores equivalent to the original scores. However, he also reported that surveys containing both positive and negative items led to increased respondent errors and researcher miscoding. In short, the inclusion of both positive and negative items in the SUS has introduced adverse effects, and since the IUS is derived from the SUS, these issues are likely to affect the IUS as well. Reverse-coded items have posed challenges for numerous self-report measures across various fields of behavioral science, raising the question of whether including both positive and negative items is truly beneficial [46, 47]. In this study, we interpret the separation of positively and negatively worded items into distinct factors primarily as a methodological artifact reflecting differential response processes, rather than as evidence of theoretically meaningful usability dimensions. Although the negatively worded items showed coherent covariance, we consider this pattern more likely to reflect systematic method variance associated with item polarity than a substantive usability construct. Nevertheless, future research could explicitly examine whether negatively worded items alone could be purposefully used to operationalize perceived lack of intervention usability, provided that such an approach is theoretically justified and psychometrically evaluated.

The SUS and subsequently the IUS were developed as tools to provide a single, comprehensive measure of perceived usability. Given this, it is essential to compare the content of these measures to proposed theoretical models of usability. The early definition describes usability as a system’s ability to bridge the gap between a user’s intended goal and the means to reach that goal [9]. A contemporary definition of usability in terms of the ISO standard extends this definition by highlighting the attributes of effectiveness, efficiency and satisfaction as key determinants of usability [11]. Furthermore, a seminal theory of usability [10] underscores the multifaceted nature of the construct by defining usability through five distinct facets: learnability, efficiency, memorability, errors, and satisfaction. Previous findings regarding the construct validity of the IUS identified some of these facets, namely, “usability” and “learnability”. A noteworthy detail from earlier studies regarding the SUS and the IUS is the tendency to name the latent factors as “learnability” and “usability”. With closer examination, the factorial incoherences and measurement paradox arise as an item that can be argued to fall under the learnability factor fails to load onto it (“I would imagine that most people would learn to use this intervention very quickly”), and the latent factor “usability” emerges separately within a measure that is supposed to represent usability as a whole. Given these measurement inconsistencies and that the IUS does not fully capture all theoretically relevant facets of usability by omitting effectiveness, efficiency, memorability, and satisfaction, the way the IUS measures usability remains theoretically ambiguous.

To address these inconsistencies, it is worth discussing whether the measurement model in the context of usability should be formative rather than reflective [48]. In reflective measurement models, it is assumed that individual items measure a common underlying construct, share a common meaning and that the observed variables are influenced by an unobserved construct. In formative measurement models, it is assumed that the measured items form the construct itself. There is a possibility that there is not a single common latent construct that is shared by all the theoretically relevant facets that we call usability; rather, we should focus on measuring the theoretically relevant facets individually and determine that they, as a whole, represent usability. However, since previous studies on the SUS and IUS use a reflective measurement model, further development of reflective models is advisable, considering both psychometric properties and theoretical frameworks. At the same time, the possibility of a formative measurement model being more suitable for conceptualizing the measurement of usability should not be overlooked.

It is plausible that the concept of usability does not transfer from the human-computer interaction (HCI) context to the context of implementing psychological interventions, and it may be unclear how incorporating this construct adds value in this domain. The risk is that we implement measures too quickly and focus on interpreting results that are unclear or lack practical value. The most robust finding in this study is that the psychometric properties of IUS differ across different psychological interventions. This raises the question of whether usability (operationalized this way) is a relevant construct to measure in the context of psychological interventions and, if so, whether it fundamentally differs from usability in the more conventional HCI context. The original application of the IUS was to assess the usability of so-called “complex” interventions; however, what constitutes a complex intervention is not exactly defined. To our knowledge, the IUS has been evaluated so far in the context of motivational interviewing [21] and two distinct cognitive‒behavioral interventions (this study). It can be argued that the goals of these interventions vary in a way that limits their direct comparability, as they are designed to address the needs of different clinical populations and conditions. Motivational interviewing is often carried out as a working method for eliciting motivation toward healthy behaviors (e.g., increasing exercising) or as a distinct intervention to treat addictions. Moreover, guided self-help and high-intensity CBT differ heavily in terms of intervention goals. Given these differences, it may be difficult to apply a single usability measure grounded in the notion of an the interventions ability to “bridge the gap between a user’s intended goal and the means to reach that goal”. Conversely, one could argue that these distinct psychological interventions share some form of common goal (e.g., improving the mental state or wellbeing of the recipient). However, this raises an important question: at what level of abstraction is usability a relevant concept to operationalize and how should intervention complexity be more precisely defined? For example, it might be easier for a professional to assess the usability of a single breathing exercise intervention in alleviating momentary anxiety rather than assessing the usability of a multi-component intervention such as CBT when treating anxiety disorders. However, this shift in focus could move attention away from complex interventions and may be impractical from a measurement perspective, given that structured intervention programs often comprise dozens of individual therapeutic actions. Taken together, the findings of this study suggest that the level of abstraction itself poses challenges for individuals in assessing how usable they find a given intervention. To follow the contemporary literature on usability, it might be beneficial to formulate a measure of usability that is more domain-specific [12] by addressing the theoretically relevant facets of usability (e.g., efficiency), specifically in the context of psychological interventions and previously recognized barriers in implementing these interventions [20]. Before this, we do not recommend the use of the IUS for assessing psychological interventions and their implementation.

If usability is intended to function as a comparative implementation construct across interventions of differing intensity and structure, its measurement must be theoretically grounded and empirically robust across those levels. Our findings suggest a need for a more explicit, theory-driven and context-dependent roadmap for measuring usability in complex psychological interventions.

First, the intended level of measurement and intervention complexity should be clearly specified, distinguishing between the usability of discrete therapeutic techniques and that of multi-component interventions. Second, domain-specific facets of usability should be identified and operationalized based on both usability theory and implementation science, such as efficiency, learnability, error proneness, and satisfaction as they manifest within clinical workflows. Third, the appropriate measurement model should be carefully considered, as usability in the context of psychological interventions may be better conceptualized as a formative rather than a reflective construct. Finally, if usability is intended to function as a comparative construct across different interventions, measurement invariance across interventions should be empirically demonstrated rather than assumed.

### Limitations

This study has limitations. First, we used a translated version of the IUS, and it is possible that nuances in the wording of individual items were altered during the translation process, which could affect respondents’ interpretation and influence the construct validity analyses. In addition, we did not conduct a formal cognitive debriefing procedure, which may have limited our ability to systematically assess how respondents interpreted individual items. Nevertheless, the translation was conducted rigorously, involving multiple independent translations and back-translation procedures. Second, there were some differences in some demographic variables between the two interventions assessed in the present study, mainly professional background, which may influence the tendency to evaluate psychological interventions. Third, the number of male respondents in both datasets was low. This can be a limitation but, on the other hand, reflects the gender disparities in these fields in general. Fourth, achieving acceptable fit for the CFA model required post hoc modifications based on correlation residuals and modification indices. Although these modifications were theoretically interpretable, such data-driven adjustments increase the risk of overfitting to sample-specific characteristics. Fifth, we did not apply formal screening for contradictory response patterns that may arise from the inclusion of reverse-worded items in the IUS. The instrument does not include established inconsistency indices or validated protocols for identifying such response patterns, which may have contributed to method effects observed in the factor structure.

## Conclusions

This study provides evidence that the psychometric properties of the IUS do not capture usability as previously suggested and that the content of the measure does not follow the theoretical foundations of usability. If the need to measure the usability of psychological interventions continues, the development of usability measures that are domain specific to psychological interventions and that follow more closely to usability theory is warranted. Afterward, the predictive validity of the measure should be monitored in relation to implementation intentions, upkeep, clinical outcomes and other appropriate outcomes to determine the measure’s practical significance. Before further theoretical refinement, domain-specific measure development, and empirical validation, we do not recommend the use of the IUS in assessing psychological interventions and to guide implementation.

## Electronic supplementary material

Below is the link to the electronic supplementary material.

Supplementary Material 1

Supplementary Material 2

Supplementary Material 3

Supplementary Material 4

Supplementary Material 5

## Data Availability

Data and analysis scripts are available upon request from the corresponding author.
