# Supplementary material for: Construct validity, reliability and measurement invariance of the intervention usability scale - insights from two psychological interventions in primary health care
Source: Implement Sci Commun. 2026 May 22;7:136. doi: 10.1186/s43058-026-00951-w (PMC13383391; doi:10.1186/s43058-026-00951-w)
Supplement: Supplementary file 1 — Supplementary Material 1 [file 43058_2026_951_MOESM1_ESM.docx]

Additional File 1: Results of the exploratory factor analysis. H^2^ = communality, com = complexity, RMSR = root mean square of the residuals, RMSEA = root mean square error of approximation (+90% CI), TLI = Tucker-Lewis Index.

|  | 2 Factors | | | | | 3 Factors | | | | | 4 Factors | | | | | |
| --- | --- | --- | --- | --- | --- | --- | --- | --- | --- | --- | --- | --- | --- | --- | --- | --- |
|  | Factor 1 | Factor 2 | H^2^ | com | | Factor 1 | Factor 2 | Factor 3 | H2 | com | Factor 1 | Factor 2 | Factor 3 | Factor 4 | H^2^ | com |
| Q1 | .71 | -.14 | .43 | 1.1 | | -.01 | -.06 | .69 | .45 | 1.0 | .00 | -.06 | .07 | .64 | .55 | 1.0 |
| Q2 | .36 | .41 | .44 | 2.0 | | .74 | .00 | -.04 | .51 | 1.0 | .72 | .05 | -.04 | .01 | .53 | 1.0 |
| Q3 | .55 | .37 | .63 | 1.7 | | .34 | .21 | .41 | .63 | 2.5 | .29 | .20 | .18 | .35 | .63 | 3.1 |
| Q4 | .04 | .71 | .53 | 1.0 | | .38 | .45 | -.04 | .51 | 2.0 | .36 | .49 | -.03 | -.01 | .52 | 1.8 |
| Q5 | .83 | -.10 | .62 | 1.0 | | .10 | -.07 | .75 | .64 | 1.1 | .10 | -.05 | .03 | .74 | .65 | 1.0 |
| Q6 | .55 | .29 | .54 | 1.5 | | .72 | -.09 | .14 | .61 | 1.1 | .70 | -.05 | -.02 | .18 | .62 | 1.1 |
| Q7 | .40 | .29 | .35 | 1.8 | | .24 | .18 | .31 | .35 | 2.6 | .01 | .00 | .81 | .02 | .68 | 1.0 |
| Q8 | .40 | .50 | .59 | 1.9 | | .78 | .07 | -.01 | .66 | 1.0 | .69 | .04 | .20 | -.06 | .67 | 1.2 |
| Q9 | .37 | .41 | .44 | 2.0 | | -.02 | .47 | .49 | .54 | 2.0 | -.02 | .46 | .07 | .45 | .54 | 2.0 |
| Q10 | -.18 | .73 | .45 | 1.1 | | .03 | .75 | -.07 | .57 | 1.0 | .00 | .74 | .05 | -.08 | .57 | 1.0 |
|  | Proportion of variance | | | | | Proportion of variance | | | | | Proportion of variance | | | | | |
|  | 27% | 23% |  | |  | 24% | 13% | 18% |  |  | 21% | 13% | 9% | 16% |  |  |
| RMSR | 0.04 |  |  | |  | 0.02 |  |  |  |  | 0.02 |  |  |  |  |  |
| RMSEA | 0.095 (0.082-0.108) | | | | | 0.072 (0.057-0.089) | | |  |  | 0.075 (0.055-0.096) | | | |  |  |
| TLI | 0.9 |  |  | |  | 0.941 |  |  |  |  | 0.937 |  |  |  |  |  |
