# Supplementary material for: Construct validity, reliability and measurement invariance of the intervention usability scale - insights from two psychological interventions in primary health care
Source: Implement Sci Commun. 2026 May 22;7:136. doi: 10.1186/s43058-026-00951-w (PMC13383391; doi:10.1186/s43058-026-00951-w)
Supplement: Supplementary file 2 — Supplementary Material 2 [file 43058_2026_951_MOESM2_ESM.docx]

Additional file 2: Correlation residuals between items (residuals >.10 in bold)

|  | Q1 | Q2 | Q3 | Q4 | Q5 | Q6 | Q7 | Q8 | Q9 | Q10 |
| --- | --- | --- | --- | --- | --- | --- | --- | --- | --- | --- |
| Q1 |  |  |  |  |  |  |  |  |  |  |
| Q2 | -.068 |  |  |  |  |  |  |  |  |  |
| Q3 | -.023 | -.027 |  |  |  |  |  |  |  |  |
| Q4 | **-.189** | -.002 | **.129** |  |  |  |  |  |  |  |
| Q5 | **.158** | -.025 | -.026 | **-.124** |  |  |  |  |  |  |
| Q6 | -.023 | .049 | -.016 | .023 | .049 |  |  |  |  |  |
| Q7 | .005 | .023 | -.031 | -.065 | -.041 | -.004 |  |  |  |  |
| Q8 | -.069 | -.012 | .029 | -.018 | -.003 | -.039 | **.124** |  |  |  |
| Q9 | .061 | -.047 | -.003 | **.130** | -.034 | -.069 | -.083 | -.069 |  |  |
| Q10 | **-.237** | .019 | .077 | .000 | **-.183** | -.051 | -.083 | .025 | **.214** |  |
