# Supplementary material for: Construct validity, reliability and measurement invariance of the intervention usability scale - insights from two psychological interventions in primary health care
Source: Implement Sci Commun. 2026 May 22;7:136. doi: 10.1186/s43058-026-00951-w (PMC13383391; doi:10.1186/s43058-026-00951-w)
Supplement: Supplementary file 3 — Supplementary Material 3 [file 43058_2026_951_MOESM3_ESM.docx]

Additional file 3: Standardized coefficients of covariance and standard errors of the item pairs allowed to correlate.

| Item pair | Standardized coefficient of covariance | Standard error |
| --- | --- | --- |
| Q1+Q4 | -0.139 | 0.049 |
| Q3+Q4 | 0.180 | 0.050 |
| Q5+Q1 | 0.190 | 0.041 |
| Q5+Q4 | -0.072 | 0.060 |
| Q8+Q7 | 0.158 | 0.045 |
| Q9+Q4 | 0.180 | 0.061 |
| Q10+Q5 | -0.145 | 0.049 |
| Q10+Q9 | 0.251 | 0.043 |
