# Supplementary material for: Construct validity, reliability and measurement invariance of the intervention usability scale - insights from two psychological interventions in primary health care
Source: Implement Sci Commun. 2026 May 22;7:136. doi: 10.1186/s43058-026-00951-w (PMC13383391; doi:10.1186/s43058-026-00951-w)
Supplement: Supplementary file 4 — Supplementary Material 4 [file 43058_2026_951_MOESM4_ESM.docx]

Additional File 4: Measurement invariance metrics by the respondent’s wellbeing service county (public healthcare organization), age, work experience, previous training in psychological interventions, and training completion status

|  | χ ^2^  (df) | CFI | RMSEA (90% CI) | SRMR | Δ CFI | Δ RMSEA | Δ SRMR | Decision |
| --- | --- | --- | --- | --- | --- | --- | --- | --- |
| **Wellbeing service county** | | | | | | | | |
| Configural invariance | 206.63 (48) *** | .985 | .085  (.073-.097) | .036 |  |  |  |  |
| Metric invariance | 182.88 (55) *** | .988 | .071  (.060-.083) | .041 | .003 | -.014 | .005 | Accept |
| Scalar invariance | 196.21 (82) *** | .989 | .055  (.045-.065) | .038 | .001 | -.016 | -.003 | Accept |
| **Age** | | | | | | | | |
| Configural invariance | 218.35 (48) *** | .982 | .090  (.078-.102) | .039 |  |  |  |  |
| Metric invariance | 177.05 (55) *** | .987 | .071  (.060-.083) | .041 | .005 | -.019 | .002 | Accept |
| Scalar invariance | 203.45 (82) *** | .987 | .058  (.048-.068) | .040 | .000 | -.013 | -.001 | Accept |
| **Work experience** | | | | | | | | |
| Configural invariance | 192.44 (48) *** | .985 | .084  (.072-.097) | .037 |  |  |  |  |
| Metric invariance | 181.32 (55) *** | .987 | .074 (.062-.086) | .042 | .002 | -.010 | .005 | Accept |
| Scalar invariance | 277.07 (82) *** | .985 | .065 (.055-.075) | .038 | -.002 | -.009 | -.004 | Accept |
| **Previous training in psychological interventions** | | | | | | | | |
| Configural invariance | 213.72 (48) *** | .983 | .087 (.076-.099) | .038 |  |  |  |  |
| Metric invariance | 187.52 (55) *** | .986 | .073 (.062-.085) | .043 | .003 | -.014 | .005 | Accept |
| Scalar invariance | 237.26 (82) *** | .984 | .065 (.055-.074) | .040 | -.002 | -.008 | -.003 | Accept |
| **Training completion status** | | | | | | | | |
| Configural invariance | 216.39 (48) *** | .984 | .086 (.075-.098) | .036 |  |  |  |  |
| Metric invariance | 157.32 (55) *** | .990 | .063 (.051-.074) | .037 | .006 | -.0023 | .001 | Accept |
| Scalar invariance | 218.32 (82) *** | .987 | .059 (.050-.069) | .037 | -.003 | -.004 | 0 | Accept |

*** p < 0.001
