# Supplementary material for: Construct validity, reliability and measurement invariance of the intervention usability scale - insights from two psychological interventions in primary health care
Source: Implement Sci Commun. 2026 May 22;7:136. doi: 10.1186/s43058-026-00951-w (PMC13383391; doi:10.1186/s43058-026-00951-w)
Supplement: Supplementary file 5 — Supplementary Material 5 [file 43058_2026_951_MOESM5_ESM.docx]

STROBE Statement—checklist of items that should be included in reports of observational studies

|  | Item No. | Recommendation | Page  No. | Relevant text from manuscript |
| --- | --- | --- | --- | --- |
| **Title and abstract** | 1 | (*a*) Indicate the study’s design with a commonly used term in the title or the abstract | 1 | The title includes information of the study design. |
|  |  | (*b*) Provide in the abstract an informative and balanced summary of what was done and what was found | 2-3 | The abstract includes information of what was done and what was found (word count: 321) |
| Introduction | | | |  |
| Background/rationale | 2 | Explain the scientific background and rationale for the investigation being reported | 6 | In Background: *“These findings* (referring to previous findings in literature)*, along with the theoretical ambiguity of the usability concept and its applicability in this context, highlight the importance of replication and further investigation.”* |
| Objectives | 3 | State specific objectives, including any prespecified hypotheses | 7 | In Background the hypotheses are stated: *1) Does the Finnish version of the IUS replicate the previous findings of a unifactorial or two-factor structure in two datasets concerning the usability of two separate cognitive‒behavioral interventions? How well do the previously recognized models fit the data and are the findings congruent between datasets?*  *2) What factor structure provides the best fit to the data, and how does it align with the theoretical basis of usability?*  *3) What is the level of measurement invariance of the Finnish version of the IUS regarding the demographic variables gathered in the datasets?* |
| Methods | | | |  |
| Study design | 4 | Present key elements of study design early in the paper | 2 | The methods are stated clearly in the Abstract as follows: “*A translated Finnish version of the IUS was administered to healthcare professionals trained in either low-intensity guided self-help (n= 921) or high-intensity face-to-face cognitive–behavioral therapy programs (n = 455). Exploratory and confirmatory factor analyses were conducted to examine the underlying structure of the IUS. Configural, metric and scalar invariance was assessed across multiple demographic subgroups. Reliability was assessed using McDonald’s Omega.”* |
| Setting | 5 | Describe the setting, locations, and relevant dates, including periods of recruitment, exposure, follow-up, and data collection | 7-10 | The setting and data collection are described under titles “Measures” and “Participants and Procedures” |
| Participants | 6 | (*a*) *Cohort study*—Give the eligibility criteria, and the sources and methods of selection of participants. Describe methods of follow-up  *Case-control study*—Give the eligibility criteria, and the sources and methods of case ascertainment and control selection. Give the rationale for the choice of cases and controls  *Cross-sectional study*—Give the eligibility criteria, and the sources and methods of selection of participants | 8 | Cross-sectional study. Under “Participants and Procedures”: “*The study participants were healthcare professionals from various professional backgrounds who had completed or were currently participating in two different training programs focused on psychological interventions for common mental health problems and disorders. All participants work or have worked in the time of training in a publicly funded mental health context in Finland. The participants were invited to a survey in which they evaluated the usability of the intervention they were trained in.”* |
|  |  | (*b*) *Cohort study*—For matched studies, give matching criteria and number of exposed and unexposed  *Case-control study*—For matched studies, give matching criteria and the number of controls per case |  |  |
| Variables | 7 | Clearly define all outcomes, exposures, predictors, potential confounders, and effect modifiers. Give diagnostic criteria, if applicable | 7 | Under Measures: *“The IUS is an adapted version of the 10-item SUS where the term ‘system’ is replaced with ‘intervention’ (21). In this study, a translated Finnish version of the IUS was evaluated.”* |
| Data sources/ measurement | 8* | For each variable of interest, give sources of data and details of methods of assessment (measurement). Describe comparability of assessment methods if there is more than one group | 8-9 | Under Participants and Procedures: *“The study participants were healthcare professionals from various professional backgrounds who had completed or were currently participating in two different training programs focused on psychological interventions for common mental health problems and disorders. All participants work or have worked in the time of training in a publicly funded mental health context in Finland. The participants were invited to a survey in which they evaluated the usability of the intervention they were trained in. The survey was sent to trainees taking part in a low-intensity guided self-help training program and in a longer training program in a higher-intensity face-to-face therapy modality.* |
| Bias | 9 | Describe any efforts to address potential sources of bias | 24-25 | Under Limitations: “*First,* *we used a translated version of the IUS, and it is possible that nuances in the wording of individual items were altered during the translation process, which could affect respondents' interpretation and influence the construct validity analyses.”*  AND “*In addition, we did not conduct a formal cognitive debriefing procedure, which may have limited our ability to systematically assess how respondents interpreted individual items.*  AND *“Furthermore, there were some differences in some demographic variables between the two interventions assessed in the present study, mainly professional background, which may influence the tendency to evaluate psychological interventions.”* AND *“Fourth, achieving acceptable fit for the CFA model required post hoc modifications based on correlation residuals and modification indices. Although these modifications were theoretically interpretable, such data-driven adjustments increase the risk of overfitting to sample-specific characteristics. Fifth, we did not apply formal screening for contradictory response patterns that may arise from the inclusion of reverse-worded items in the IUS. The instrument does not include established inconsistency indices or validated protocols for identifying such response patterns, which may have contributed to method effects observed in the factor structure”.* |
| Study size | 10 | Explain how the study size was arrived at | 9-10 | Under Dataset 1: Usability of guided self-help: *“A total of 921 (15%) respondents out of the 6 335 invited participants were enrolled in the survey”* AND under Dataset 2: Usability of face-to-face cognitive-behavioral therapy: *“A total of 455 respondents (35%) out of 1 308 invited participants were enrolled in the survey.”* |

Continued on next page

| Quantitative variables | 11 | Explain how quantitative variables were handled in the analyses. If applicable, describe which groupings were chosen and why | 10-14 | Information on how the quantitative variables were handled is included in the Statistical analyses –section. |
| --- | --- | --- | --- | --- |
| Statistical methods | 12 | (*a*) Describe all statistical methods, including those used to control for confounding | 10-14 | Information of the statistical methods are included under Statistical analyses. Controlling for confounders was not applicable for this study. |
|  |  | (*b*) Describe any methods used to examine subgroups and interactions | 19-20 | No subgroups or interactions were analysed in the study. Rather measurement invariance is described under Step 3: Measurement invariance. |
|  |  | (*c*) Explain how missing data were addressed | 10 | Under Statistical analyses: “*For all analyses, we used listwise deletion, meaning that any case (row) with missing data for the variable needed for a given analysis was removed.”* |
|  |  | (*d*) *Cohort study*—If applicable, explain how loss to follow-up was addressed  *Case-control study*—If applicable, explain how matching of cases and controls was addressed  *Cross-sectional study*—If applicable, describe analytical methods taking account of sampling strategy | 9-10 | Cross-sectional study. Under Dataset 1: Usability of guided self-help: “*The GSH data were split into exploratory and confirmatory datasets via stratified sampling to carry out the necessary analyses regarding the construct validity of the measure. The following variables were used to guide the stratified sampling process to ensure representativeness and balance across the subsets created: gender, age, profession and education level. To confirm that the background variables were balanced across the exploratory and confirmatory datasets, proportional distributions of gender, age, profession, and education were calculated for each subset. These proportions were compared to verify representativeness and ensure the integrity of the stratification process. The random seed was set to ‘123’ to allow for exact replication of the split.”* |
|  |  | (*e*) Describe any sensitivity analyses | 16 | Under Step 2: CFA with the remaining data from dataset 1: *“We attempted the same CFA with several allowed covariances. We allowed the item pairs [4,1], [4,3], [5,1], [5,4], [8,7], [9,4], [10,5] and [10,9] to correlate.”* |
| Results | | | | |
| Participants | 13* | (a) Report numbers of individuals at each stage of study—eg numbers potentially eligible, examined for eligibility, confirmed eligible, included in the study, completing follow-up, and analysed | 9-10 | Under Dataset 1: Usability of guided self-help: *“A total of 921 (15%) respondents out of the 6 335 invited participants were enrolled in the survey and under”* Dataset 2: Usability of face-to-face cognitive-behavioral therapy: *“A total of 455 respondents (35%) out of 1 308 invited participants were enrolled in the survey.”* |
|  |  | (b) Give reasons for non-participation at each stage | NA | NA: we report invited participants and those who took part. We have no information on reasons, why people did not respond to the surveys. |
|  |  | (c) Consider use of a flow diagram | NA | Participant flow can be unambiguously described via text. See item 13. |
| Descriptive data | 14* | (a) Give characteristics of study participants (eg demographic, clinical, social) and information on exposures and potential confounders | 10 | In Table 1: Demographic characteristics of the study samples. |
|  |  | (b) Indicate number of participants with missing data for each variable of interest | 10 | All missing data was deleted listwise. |
|  |  | (c) *Cohort study*—Summarise follow-up time (eg, average and total amount) | NA | NA |
| Outcome data | 15* | *Cohort study*—Report numbers of outcome events or summary measures over time |  |  |
|  |  | *Case-control study—*Report numbers in each exposure category, or summary measures of exposure |  |  |
|  |  | *Cross-sectional study—*Report numbers of outcome events or summary measures | *15-17* | We consider summary measures the fit indices of each factor analysis. Under Step 1: EFA with split dataset 1*: “Compared with the two-factor solution, the three-factor solution exhibited better overall fit, with an RMSR of 0.02, RMSEA of 0.072, and TLI of 0.941.”* Under Step 2: CFA with the remaining data from dataset 1*: “A three-factor CFA converged with a statistically significant χ ^2^ value (χ ^2^=198.052, df= 32, p<0.001). CFI (=0.948), TLI (=0.927) and SRMR (=0.077) were found close to satisfactory levels, whereas RMSEA (=0.143, 90% CI 0.124-0.163) was found to be inflated.”* Under Step 4: CFA on Dataset 2: “*A CFA model converged with a significant χ^2^ value (χ^2^=116.974, df=24, p<.001). The RMSEA was inflated (=0.109, 90% CI [0.088, 0.131]), and the TLI was below adequate (=0.917). CFI was 0.956, and SRMR was 0.055.”* |
| Main results | 16 | (*a*) Give unadjusted estimates and, if applicable, confounder-adjusted estimates and their precision (eg, 95% confidence interval). Make clear which confounders were adjusted for and why they were included | NA | All relevant estimates are described under Item 15. |
|  |  | (*b*) Report category boundaries when continuous variables were categorized | NA | No continuous variables were categorized. |
|  |  | (*c*) If relevant, consider translating estimates of relative risk into absolute risk for a meaningful time period | NA | Not relevant. |

Continued on next page

| Other analyses | 17 | Report other analyses done—eg analyses of subgroups and interactions, and sensitivity analyses | 16 | Under Step 2: CFA with the remaining data from dataset 1: *“We attempted the same CFA with several allowed covariances. We allowed the item pairs [4,1], [4,3], [5,1], [5,4], [8,7], [9,4], [10,5] and [10,9] to correlate.”* |
| --- | --- | --- | --- | --- |
| Discussion | | | | |
| Key results | 18 | Summarise key results with reference to study objectives | 18 | Under discussion, 2^nd^ paragraph: “*The two-factor structure identified in the prior study of the IUS (21) was not replicated in this study. Additionally, our analysis does not support either the unifactorial or two-factor structures previously recognized in studies regarding the SUS, from which the IUS was adapted (17).”* |
| Limitations | 19 | Discuss limitations of the study, taking into account sources of potential bias or imprecision. Discuss both direction and magnitude of any potential bias | 24-25 | Limitations are stated under Limitations. |
| Interpretation | 20 | Give a cautious overall interpretation of results considering objectives, limitations, multiplicity of analyses, results from similar studies, and other relevant evidence | 25 | Interpretation of the results is summarized under Conclusions: “*This study provides evidence that the psychometric properties of the IUS do not capture usability as previously suggested and that the content of the measure does not follow the theoretical foundations of usability. If the need to measure the usability of psychological interventions continues, the development of usability measures that are domain specific to psychological interventions and that follow more closely to usability theory is warranted. Afterward, the predictive validity of the measure should be monitored in relation to implementation intentions, upkeep, clinical outcomes and other appropriate outcomes to determine the measure’s practical significance. Before further theoretical refinement, domain-specific measure development, and empirical validation, we do not recommend the use of the IUS in assessing psychological interventions and to guide implementation.”* |
| Generalisability | 21 | Discuss the generalisability (external validity) of the study results | 19 | Especially mentioned under Discussion: “*However, the factor structure that best fit the GSH dataset did not replicate the second fCBT dataset, suggesting that the construct validity of the IUS may differ depending on the intervention being assessed”* |
| Other information | |  | | |
| Funding | 22 | Give the source of funding and the role of the funders for the present study and, if applicable, for the original study on which the present article is based | 25-26 | Under Declarations & Funding |

*Give information separately for cases and controls in case-control studies and, if applicable, for exposed and unexposed groups in cohort and cross-sectional studies.

**Note:** An Explanation and Elaboration article discusses each checklist item and gives methodological background and published examples of transparent reporting. The STROBE checklist is best used in conjunction with this article (freely available on the Web sites of PLoS Medicine at http://www.plosmedicine.org/, Annals of Internal Medicine at http://www.annals.org/, and Epidemiology at http://www.epidem.com/). Information on the STROBE Initiative is available at www.strobe-statement.org.
